# Antibiotrophy: Key Function for Antibiotic-Resistant Bacteria to Colonize Soils—Case of Sulfamethazine-Degrading *Microbacterium* sp. C448

**DOI:** 10.3389/fmicb.2021.643087

**Published:** 2021-03-26

**Authors:** Loren Billet, Stéphane Pesce, Nadine Rouard, Aymé Spor, Laurianne Paris, Martin Leremboure, Arnaud Mounier, Pascale Besse-Hoggan, Fabrice Martin-Laurent, Marion Devers-Lamrani

**Affiliations:** ^1^AgroSup Dijon, INRAE, Université de Bourgogne Franche-Comté, Agroécologie, Dijon, France; ^2^INRAE, UR RiverLy, Villeurbanne, France; ^3^Université Clermont Auvergne, CNRS, Sigma Clermont, Institut de Chimie de Clermont-Ferrand, Clermont-Ferrand, France

**Keywords:** sulfonamide, microbial ecotoxicology, bacterial community invasion, soil, antibiotic biodegradation

## Abstract

Chronic and repeated exposure of environmental bacterial communities to anthropogenic antibiotics have recently driven some antibiotic-resistant bacteria to acquire catabolic functions, enabling them to use antibiotics as nutritive sources (antibiotrophy). Antibiotrophy might confer a selective advantage facilitating the implantation and dispersion of antibiotrophs in contaminated environments. A microcosm experiment was conducted to test this hypothesis in an agroecosystem context. The sulfonamide-degrading and resistant bacterium *Microbacterium* sp. C448 was inoculated in four different soil types with and without added sulfamethazine and/or swine manure. After 1 month of incubation, *Microbacterium* sp. (and its antibiotrophic gene *sadA*) was detected only in the sulfamethazine-treated soils, suggesting a low competitiveness of the strain without antibiotic selection pressure. In the absence of manure and despite the presence of *Microbacterium* sp. C448, only one of the four sulfamethazine-treated soils exhibited mineralization capacities, which were low (inferior to 5.5 ± 0.3%). By contrast, manure addition significantly enhanced sulfamethazine mineralization in all the soil types (at least double, comprised between 5.6 ± 0.7% and 19.5 ± 1.2%). These results, which confirm that the presence of functional genes does not necessarily ensure functionality, suggest that sulfamethazine does not necessarily confer a selective advantage on the degrading strain as a nutritional source. 16S rDNA sequencing analyses strongly suggest that sulfamethazine released trophic niches by biocidal action. Accordingly, manure-originating bacteria and/or *Microbacterium* sp. C448 could gain access to low-competition or competition-free ecological niches. However, simultaneous inputs of manure and of the strain could induce competition detrimental for *Microbacterium* sp. C448, forcing it to use sulfamethazine as a nutritional source. Altogether, these results suggest that the antibiotrophic strain studied can modulate its sulfamethazine-degrading function depending on microbial competition and resource accessibility, to become established in an agricultural soil. Most importantly, this work highlights an increased dispersal potential of antibiotrophs in antibiotic-polluted environments, as antibiotics can not only release existing trophic niches but also form new ones.

## Introduction

Antibiotics and their residues deserve specific attention as pollutants of emerging concern. Most are slightly and partially metabolized in humans or animals prior to excretion (Lamshöft et al., 2007; Hawkshead, 2008), resulting in heavy contamination of wastewater and livestock manure (Kümmerer, 2001; Watkinson et al., 2007; Feng et al., 2017), where they can reach concentrations of up to micrograms per liter (Peng et al., 2006; Rodriguez-Mozaz et al., 2020) and milligrams per kilogram (Haller et al., 2002; Cid et al., 2018), respectively. The disposal processes applied to these matrices lack efficiency, and so the discharge of wastewater into aquatic ecosystems or manure spread on soils, a common agricultural practice for nutrient recycling and soil fertilization, contributes to the release of antibiotics into different environmental compartments (Watkinson et al., 2007; Heuer et al., 2011; Ghirardini et al., 2020).

By their mode of action and their targets, antibiotics have several ecotoxicological effects on environmental bacteria. First, the direct biocidal action of an antibiotic can alter microbial communities and disturb their function supporting ecosystem services (Costanzo et al., 2005; Ding and He, 2010; Hammesfahr et al., 2011; Pinna et al., 2012; Proia et al., 2013; Cycoń et al., 2019). For instance, tetracycline can alter the denitrification process of soil microbial communities (Shan et al., 2018), while sulfamethazine was shown to negatively impact the phosphatase activity and the respiration of soil microflora (Liu et al., 2009). Second, exposure to antibiotics can induce an adaptive response of microbial communities, notably through the development and spread of antibiotic-resistant bacteria and genes. Numerous studies have shown a positive correlation between antibiotic contamination and the occurrence of antibiotic-resistance genes in ecosystems receiving the above polluted matrices (Hawkshead, 2008; Zhang et al., 2009; Bengtsson-Palme and Larsson, 2016; Bengtsson-Palme et al., 2018). Contamination of ecosystems by antibiotics is thus a major health concern, as these environmental microbial communities constitute reservoirs of resistance that might potentially be transferred to pathogenic bacteria (Perry and Wright, 2013; Zrimec, 2020).

The adaptive capacities of environmental microorganisms toward organic pollutants are not only limited to the increased tolerance or resistance but also include the development of catabolic pathways (Pesce et al., 2020). In this way, the long-term exposure of microbial communities to these substances can lead to the emergence of bacteria able to use them as nutrient sources (Copley, 2000; Janssen et al., 2005). Although the pesticide-degrading function has been extensively studied (Behki and Khan, 1986; Rani and Lalithakumari, 1994; Aislabie et al., 1997; Gisi et al., 1997), the existence of antibiotic-degrading bacteria was reported more recently (Barnhill et al., 2010; Xin et al., 2012; Topp et al., 2013; Leng et al., 2016; Crofts et al., 2018). The low environmental concentrations of antibiotics compared to pesticides, together with their biocidal effects on many microorganisms, have probably slowed down the development, emergence, and spread of antibiotic-degrading functions. However, the likelihood of such emerging abilities has risen with the increasing concentrations of antibiotics in the environmental compartments along with the decreasing sensitivity of microbial communities. The latest effect is due to the spread of a wide range of antibiotic resistances (Stoll et al., 2012; Bengtsson-Palme et al., 2018) and/or to phenotypic adaptation following chronic exposure to antibiotics (Schmitt et al., 2005; Corona and Martinez, 2013).

In the last decade, a handful of antibiotic-resistant bacteria able to use some antibiotics as nutritive sources were isolated from various environmental matrices repeatedly exposed to relatively high concentrations of antibiotics as agricultural soil (Topp et al., 2013), membrane bioreactor (Bouju et al., 2012), and activated sewage sludge (Lin et al., 2015). The degradative function, termed “antibiotrophy” (Woappi et al., 2016), is specific to an antibiotic or a class of antibiotics, such as β-lactams (Lin et al., 2015; Crofts et al., 2018) or erythromycin (Gao et al., 2015). The case of sulfonamide is currently the most documented (Bouju et al., 2012; Tappe et al., 2013; Topp et al., 2013; Ricken et al., 2017; Kim et al., 2019).

Among antibiotics, sulfonamides are synthetic bacteriostatic compounds largely used in veterinary and human medicine (Shah et al., 2013). They act as competitive inhibitors of the dihydropteroate synthase involved in the synthesis pathway of nucleic acids (Brown, 1962). The most documented resistance mechanism relies on four genes (*sul1*, *sul2*, *sul3*, and *sulA*), coding for dihydropteroate synthase isoproteins insensitive to sulfonamide (Wise and Abou-Donia, 1975; Yun et al., 2012). Both sulfonamides and *sul* genes are widely detected in different environmental matrices such as livestock manure, surface water, and river sediment (Pei et al., 2006; McKinney et al., 2010; Stoll et al., 2012). The dispersion potential of these plasmid-borne resistant genes is enhanced by their common association with integrons (Antunes et al., 2005; Wei et al., 2018), which also contain a variety of resistance genes toward many kinds of biocides (e.g., disinfectants, metals, and antibiotics). Exposure to these biocides can therefore lead to a co-selection of sulfonamide resistances (Pei et al., 2006; Luo et al., 2010; McKinney et al., 2010; Hoa et al., 2011; Stoll et al., 2012). Sulfonamide antibiotrophy is a recent discovery. Reports on environmental sulfonamide-degrading bacteria are still few but increasing. Those already described originate from different geographically distant matrices, but are phylogenetically close, and their sulfonamide degradative pathway relies on the same cluster of genes (Ricken et al., 2017; Kim et al., 2019). It composed of two flavin-dependent monooxygenases (*sadA* and *sadB*) and one flavin mononucleotide reductase (*sadC*). Although genetic and metabolic studies have been conducted on isolated sulfonamide antibiotrophs, information is lacking on the ecological impact of this function for bacterial carriers in complex environmental bacterial communities. Manure amendment on soils, known not only to increase the fertility of soils but also to introduce biological contaminants and chemical pollutants, may also be involved in the environmental dispersion of antibiotrophy (Heuer et al., 2011; Tappe et al., 2013; Chen et al., 2019).

At first glance, the development of antibiotic degradation capacities might seem environmentally beneficial by increasing the self-purifying potential of ecosystems, contributing to the natural attenuation of their contamination by antibiotics. Likewise, it could be perceived as a bioremediation tool that could improve the removal of antibiotics from contaminated matrices before their discharge into the environment (Hirth et al., 2016; Sharma et al., 2018; Kumar et al., 2019; Silva et al., 2020). However, given that currently known antibiotrophs are also antibiotic-resistant, the question arises of whether antibiotrophy could lend them a selective advantage and so facilitate their implantation and dispersion in the environment.

We conducted a microcosm study to investigate the mechanisms driving the emergence and development of antibiotrophy in agricultural soils. Specifically, we investigated whether an allochthonous bacterium harboring both sulfonamide resistance and degradation genes could become established within natural microbial communities in soils subjected to both sulfonamide contamination and manure amendment. For this purpose, we considered four different soil types with and without added sulfamethazine (SMZ) and/or swine manure, and with and without inoculation with the sulfonamide-degrading and resistant bacterial strain, *Microbacterium* sp. C448. This model bacterium was isolated from experimental soils exposed for 10 years to an annual increasing dose of SMZ ranging from 1 to 10 mg kg^–1^ (Topp et al., 2013). It presents the advantage of being well-characterized genetically and physiologically (Martin-Laurent et al., 2014). To assess the establishment of *Microbacterium* sp. C448 in soil communities, its abundance was measured, after 1 month of incubation, by qPCR assay targeting its *groL* gene sequence. The resulting effect of applied treatments on the soil community’s genetic potential for sulfonamide degradation was assessed through the quantification of the *sadA* gene sequence. To determine whether the degradation potential was expressed, we measured the mineralization kinetics of ^14^C-SMZ during the incubation period and quantified the remaining extractable fraction of SMZ at the end of the incubation period. Finally, we assessed the effects of the eight treatments on the soil microbial community structure and diversity by Illumina 16S rDNA sequencing.

## Materials and Methods

### Collection and Analyses of Soils and Manure

Soils and manure were collected in the Bourgogne–Franche–Comté region of France in November 2018. Four soils, A, B, C, and D, selected for their different textures, were sampled from the upper 20 cm of different agricultural fields, air-dried, sieved (mesh size 5 mm), and stored in airtight plastic bags at 4°C until use. Soil A originated from an agricultural field frequently flooded by the nearby Saône River. Soil B came from the same plot but was less often flooded owing to its higher altitude. Soil C originated from the experimental farm of Epoisses. Soil D was sampled near a hog nursery and had received manure 6 months before the sampling. The pedological variables of the soils (pH, ion retention, grain size, and water content at pF 2.7) are given in Supplementary Table 1. Manure was collected from the covered pit of a hog nursery and stored in plastic bottles at 4°C until use. SMZ quantification was performed on soil and manure samples (Supplementary Table 1). SMZ concentration reached 0.94 μg L^–1^ in manure.

### Microcosm Experiment Setup

The experiment consisted in incubating soil treated or not with manure, contaminated or not with SMZ, and inoculated or not with *Microbacterium* sp. C448. Considering eight conditions and five replicates per condition, 40 microcosms for each soil were prepared as described below. Microcosms containing 50 g dry weight (DW) of soils were prepared in glass bottles closed with air-permeable lids made of gauze and cotton (Supplementary Figure 1). They were incubated for 1 week at 20°C (±1°C) to gently activate the native soil microorganisms. Soil microcosms were humidified up to 80% of their water content at pF 2.7. To inoculate microcosms with *Microbacterium* sp. C448, the strain was first grown in LB-Miller liquid medium at 28°C under 130 rpm agitation. The grown cells were collected in their exponential phase (OD_600*nm*_ ∼ 1 AU) and washed twice with NaCl 0.9%. SMZ sodium salt [SMZ, CAS number: 1981-58-4, 4-amino-*N*-(4,6-dimethyl-2-pyrimidinyl)benzenesulfonamide, Sigma-Aldrich, France] was diluted to 100 mg ml^–1^ in water and filtered (0.2 μm). The manure was incubated overnight at room temperature (20 ± 1°C). Treatments were prepared a few minutes before their application on soils to reach nominal concentrations of 10^4^ cells of *Microbacterium* sp. C448 g^–1^ dry soil, 100 mg of SMZ kg^–1^ dry soil, and 13 ml of manure kg^–1^ dry soil [eight treatments, five replicates (Supplementary Figure 1)]. The microcosms were incubated at room temperature in the dark for 1 month and maintained at a stable humidity by regular watering (80% of their water content at pF 2.7). At the end of the incubation, we subsampled each microcosm for DNA extraction and further molecular analyses.

### SMZ Fate Monitoring

#### SMZ Mineralization Monitoring

For the soil treated with SMZ, the mineralization SMZ was monitored over the time of incubation by radiorespirometry. To do so, the SMZ-treated microcosms were duplicated, with one series being exposed to ^12^C-SMZ and another one being exposed to a mixture of ^12^C- and ^14^C-SMZ (Supplementary Figure 1). These last ones were treated with 0.11 μCi of ^14^C-ring-labeled SMZ (^14^C-SMZ, CAS number: 57-68-1, radiochemical purity: >98%, specific radioactivity: X MBq mmol^–1^) and placed in closed respirometer jars. ^14^CO_2_ released from the ^14^C-SMZ degradation was trapped in 5 ml of 0.2 M NaOH solution and regularly analyzed by liquid scintillation counting using ACS II scintillation fluid (Amersham).

#### SMZ Quantification

The initial concentration of SMZ in the matrices used in this study (soil and manure) as well as the SMZ remaining in the soil microcosms at the end of the incubation was measured as described below. To Nalgene PPCO 50-ml centrifuge tubes (checked for lack of SMZ sorption capacity) were added 6.0000 ± 0.0005 g of each previously dried and crushed SMZ-contaminated soil sample and 20 ml of MeOH/H_2_O 4/1 (v/v) mixture. The tubes were stirred for 18 h using an orbital shaker (Heidolph Reax) at 50 rpm and room temperature. After centrifuging (12,500 × *g* for 15 min), the supernatant was filtered on a 0.45-μm syringe PVDF filter (after checking for lack of sorption) before injecting on HPLC. The four soils were spiked with standard SMZ solutions at different concentrations and underwent the same protocol. The recovery percentages ranged between 79 ± 1% and 93 ± 2% according to the soil. The antibiotic concentrations of soil liquid extracts were determined by HPLC on an Agilent 1,100 apparatus (Courtaboeuf, France) equipped with a reverse-phase column (C18 Zorbax Eclipse Plus column, 75 mm × 4.6 mm, 3.5 μm) at 22°C and a diode array detector set at λ = 260 and 298 nm. The mobile phase composed of aqueous H_3_PO_4_ (0.01% v/v, pH = 2.9) (A) and acetonitrile (B) at a flow rate of 1 ml min^–1^. Gradient (linear): 0–5 min: 2% B; 5–8 min: 2–30% B; 8–10 min: 30–90% B; 10–10.5 min: 90–100% B; 10.5–11.5 min: 100% B; 11.5–13 min: 100–2% B. Injection volume: 10 μl. Each sample was analyzed twice (technical duplicate). Some samples were also treated with the LC/ESI-MS protocol (below) to check the SMZ concentrations. These were diluted 1/1000 before LC/MS analysis.

For the non-contaminated soil samples, the protocol was the same except that 50 μl of a 5000 μg L^–1^ SMZ-d_4_ (LCG standards) solution was added to each centrifuge tube. After centrifuging, the supernatant was concentrated in a Speed-Vac vacuum concentrator heated at 35°C. The residue was dissolved in 10 ml of distilled water. After adjusting the pH to 3.8 ± 0.2, samples were concentrated 40-fold on Oasis HLB 500-mg cartridges (Waters^TM^) according to the manufacturer’s recommendations (elution: 10 ml methanol). The antibiotic concentrations were determined by LC/ESI-MS on a Thermo Scientific UHPLC Ultimate 3000 RSLC coupled with an Orbitrap Q-Exactive analyzer. The analyses were carried out in positive mode. The UHPLC was equipped with a Luna Omega Polar C18 column; 100 × 2.1 mm; 1.6 μm (Phenomenex) at 30°C with acetonitrile gradient +0.1% formic acid (Solvent A) and water +0.1% formic acid (Solvent B): 0–2.5 min: 30–64.5% A (linear); 2.5–2.6 min: 64.5–99% A (linear); 2.6–5 min: 99% A; 5–5.1 min: 99–30% A; 5.1–8 min: 30% A. Flow rate of 0.45 ml min^–1^. For the mass spectrometer, gaseous N_2_ was used as nebulizer gas (50 A.U.). The spray voltage was 3.0 kV.

### Soil DNA Extraction and Real-Time PCR

DNA was extracted from 250 mg of soil from each sample using the DNeasy PowerSoil-htp 96-well DNA isolation kit (Qiagen, France) following the manufacturer’s instructions and stored at −20°C.

Real-time PCR reactions were carried out in a ViiA7 (Life Technologies, United States) in a 15-μl reaction volume. For *sadA* and *rss* genes, the mixture contained 7.5 μl of Takyon MasterMix (Eurogentec, France), 1 μM of each primer (Supplementary Table 2), 250 ng of T4 gene 32 (QBiogene, France), and 3 ng of DNA. The quantification of *Microbacterium* sp. C448’s *groL* required a specific probe. The reaction mixture contained 7.5 μl of Takyon Low ROX Probe 2X MasterMix dTTP blue (Eurogentec, France), 400 nM of each primer, 200 nM of the probe (Supplementary Table 2), 250 ng of T4 gene 32, and 3 ng of soil DNA. Each real-time PCR assay was performed twice independently. Standard curves were obtained using serial dilutions of linearized plasmids containing the appropriate cloned target genes from bacterial strains. The qPCR efficiencies ranged between 80 and 100%. Inhibition was assessed for each sample in accordance with ISO17601 (2016). No inhibition was detected in any case. The thermal cycling was performed as follows: 95°C for 10 min, 40 cycles of 95°C for 30 s, annealing (Supplementary Table 2) for 30 s, and 30 s at 60°C. Relative abundances were obtained by normalizing the copy number of genes *sadA* and *groL* with bacterial 16S rRNA gene *rss* copies. The detection limit was 50 copies/ng of DNA for *sadA* and *groL* and 5,000 copies/ng of DNA for *rss*. The threshold expressed by 1,000 copies of *rss* was determined by dividing the detection limit of the gene by the lowest quantification value of *rss*.

### 16S rDNA Sequencing and Bioinformatic Analysis

The V3–V4 hypervariable region of the bacterial 16S rRNA gene was amplified using a two-step PCR and sequenced on MiSeq (Illumina, 2 × 250 bp) as previously described (Spor et al., 2020).

A Jupyter Notebook developed in-house was used to analyze the sequence data. Briefly, sequences were assembled using PEAR (Zhang et al., 2014) with default settings. Further quality checks were conducted using the QIIME pipeline (Caporaso et al., 2010) and short sequences were removed (<400 bp). Reference-based and *de novo* chimera detection together with clustering in OTUs were performed using VSEARCH (Rognes et al., 2016) and Greengenes’ representative set of 16S rRNA sequences as the reference database. The identity thresholds were set at 94%. Representative sequences for each OTU were aligned using Infernal (Nawrocki and Eddy, 2013) and a 16S rRNA phylogenetic tree was constructed using FastTree (Price et al., 2010). Taxonomy was assigned using RDP Classifier (Wang et al., 2007) and the latest released Greengenes database (v.05/2013; McDonald et al., 2012). α-Diversity metrics (PD whole tree, species richness, and equitability index) were determined from rarefied OTU tables of 25,700, 24,500, 28,300, and 23,100 sequences per sample for soils A, B, C, and D, respectively. Sequences were deposited in the SRA at NCBI under the accession number SUB8712901.

### Statistical Analyses

Statistical analyses were carried out using RStudio statistical software (version 1.2.5033). For each soil, we built an *ad hoc* analysis of variance (ANOVA) model to determine the effects of treatments on gene abundances and on the alpha diversity indices of bacterial communities. Normality and homogeneity of the residual distribution were inspected and log10-transformations were performed when necessary. Differences in response variable related to the treatment (antibiotic treatment, manure amendment, and strain inoculation) were detected using the following model:

$$\begin{array}{rcl} Y_{ijkl} & = & \text{μ}+A_{i}+T_{j}+I_{k}+A:T_{ij}+A:I_{ik}\\ & & +T:I_{jk}+A:T:I_{ijk}+{\text{ε}}_{ijkl}, \end{array}$$

where *Y*_*ijkl*_ is the response variable, *A* is the fixed effect of manure amendment (*i* = 1,2), *T* is the fixed effect of sulfamethazine treatment (*j* = 1,2), *I* is the fixed effect of inoculation by *Microbacterium* sp. C448 (*k* = 1,2), and *ε_*ijkl*_* are the residuals.

Tukey’s test was performed to detect significant pairwise differences for each treatment (significance threshold set at *p* < 0.05).

The OTU table was also filtered to keep the dominant OTUs (relative abundance > 0.1%). For each soil, the OTUs that were significantly impacted by treatments were detected with the previous linear model. We opted for a custom model analysis to take into account the relative complexity of our experimental design that cannot be handled by classical analysis packages such as edgeR or DESeq2. A false discovery rate procedure was used to decrease substantially the probability of detecting false positives. The FDR significance threshold was set at 0.05.

## Results and Discussion

### The Soils, Regardless of Manure Amendment, Did Not Have Innate Antibiotrophic Potential or Activity

**FIGURE 1:**
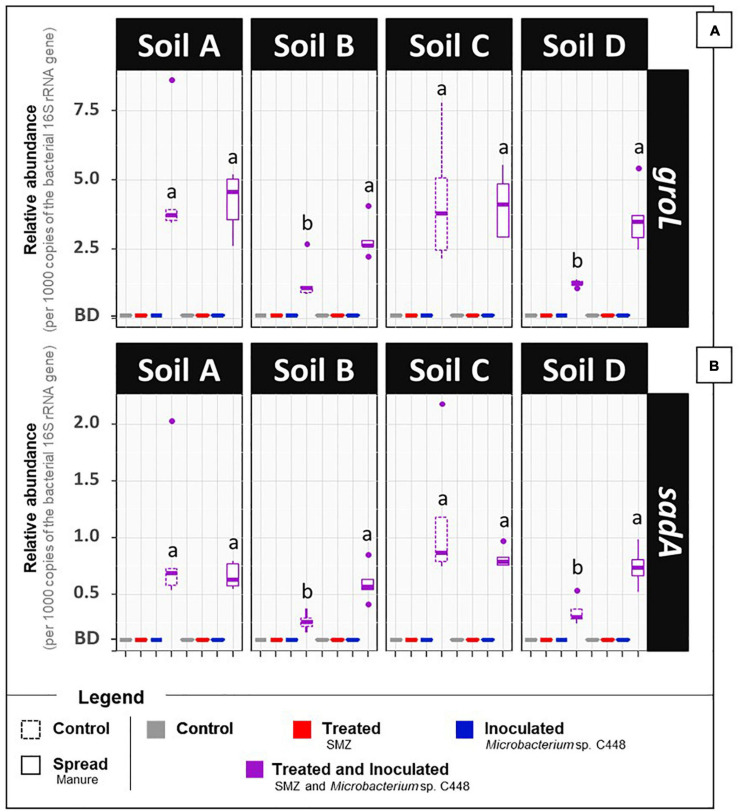
Relative abundances of *groL* **(A)** and *sadA* **(B)** gene sequences in the soil microcosms (four types of soil, A, B, C, and D, amended or not with manure, treated or not with SMZ, and inoculated or not with *Microbacterium* sp. C448). After 1 month of incubation, gene sequence abundances were measured by quantitative PCR and expressed per 1000 copies of the bacterial 16S rRNA gene *rss* (Tukey’s test, *n* = 5, *p* < 0.05). BD: below the detection limit (0.1 copy per 1,000 16S rRNA gene).

**FIGURE 2:**
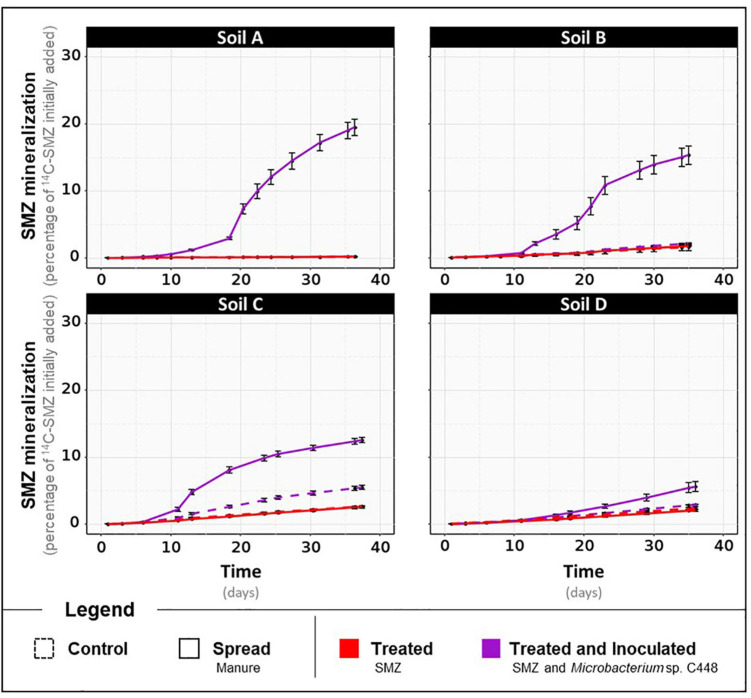
Kinetics of ^14^C-SMZ mineralization throughout the incubation for the four different types of soils, A, B, C, and D, treated with SMZ, amended or not with manure, and inoculated or not with *Microbacterium* sp. C448. Results are expressed in percentage of initially added ^14^C-SMZ.

After a 1-month incubation period, the antibiotrophic gene *sadA* was not detected in the four soils even when they were amended with manure (Figure 1B, detection limit of 0.1 *sadA* copies per 1,000 *rss*). Despite the exposure to SMZ, its abundance remained below the detection limit. This strongly suggests that the microbial communities of the four soils and manure do not initially possess bacteria carrying *sadA*, although some of them were previously exposed to low concentrations of SMZ (Supplementary Table 3). *sadA* has only appeared in recent literature on sulfonamide-degrading bacteria isolated from environmental compartments after high and long-term exposure to sulfonamide antibiotics, such as bioreactor membranes (Bouju et al., 2012), active sludges (Reis et al., 2014), or manure-amended soils (Tappe et al., 2013; Chen et al., 2019). However, the lack of detection of the key gene coding for the first enzyme of the only currently established SMZ antibiotrophy pathway cannot exclude the possibility of antibiotrophic activity catalyzed by other still unknown enzymes. We also measured the antibiotrophic potential activity. These analyses relied on a similar microcosm experiment differing only by the addition of ^14^C-labeled SMZ to monitor the mineralization of its phenyl ring. The mineralization rates obtained for soils, regardless of manure amendment and SMZ treatment modalities, did not exceed 3% of initially added ^14^C-SMZ (Figure 2). Although an alternative low-efficiency SMZ-antibiotrophic pathway can explain this slight observed mineralization, it can be reasonably assumed that it results from residual mineralization. Nonspecific biotic and abiotic mechanisms can partially break complex molecules (Gold et al., 2000; Mitchell et al., 2015), promoting fortuitous biological degradation. Moreover, the mineralization rates did not increase during the 1 month of exposure of native soil microbial communities to a high concentration of SMZ. We note that the experimental conditions were insufficient to guarantee enhancement and development of antibiotrophic abilities.

### *Microbacterium* sp. C448 Needs SMZ to Become Established in the Soil Community

After a 1-month incubation period, quantification of the *Microbacterium* sp. C448 through the estimation of the abundance of its *groL* gene sequence (Figure 1A) indicated that it could establish in the four tested soils but only under SMZ exposure. The relative abundance of the strain was relatively homogenous in the four treated soils (from 1.3 ± 0.1 to 4.6 ± 1.6 *groL* copies per 1,000 copies of the 16S rRNA gene rrs), despite their different biotic and abiotic properties. Adding manure had either no impact (soils A and C) or a relatively limited one (soils B and D, *n* = 5, *p* < 0.05) on the relative abundance of the *groL* gene sequence. Extrapolating *groL* quantification and assuming a soil DNA extraction yield of 1, at least 10^6^ cells of *Microbacterium* sp. C448 per gram of dry soil were counted at the end of incubation in the SMZ-treated soils inoculated with 10^4^ cells per gram of dry soil, indicating growth. Conversely, in the SMZ-untreated soils, *groL* gene sequences were below the detection limit of 0.1 *groL* per 1,000 *rss*, suggesting that *Microbacterium* sp. C448, initially absent, did not become established in these conditions. This indicates a low competitiveness for this strain, which possesses a low invasion potential under SMZ-free conditions and highlights the mandatory presence of the antibiotic for its establishment. SMZ may therefore be the main environmental driver controlling the efficiency of the establishment of *Microbacterium* sp. C448, at least in our experimental conditions. Given that this strain is also known to resist and degrade other sulfonamides such as sulfamethoxazole, sulfadiazine, or sulfadimethoxine (Martin-Laurent and Topp, unpublished data), this effect might hold for a wider range of antibiotics in this family.

Two main hypotheses could explain this selective advantage conferred by SMZ. The first is that the antibiotic may be used as a nutrient source exclusively dedicated to *Microbacterium* sp. C448, thus playing a major role in ecological selectivity (Nishiyama et al., 1993; Karpouzas and Walker, 2000; Hong et al., 2007). Specialized microorganisms, when they are the only ones possessing the required catabolic enzymes to mineralize selected xenobiotics, are able to establish and perform mineralization even if they are initially scant in the microbial community (van Veen et al., 1997). The second hypothesis is that SMZ may release trophic niches by biocidal action (Pinna et al., 2012; Reichel et al., 2015), thus reducing negative interactions such as trophic interactions or competition (van Elsas et al., 2012). These two non-exclusive hypotheses may explain why SMZ facilitates the establishment of the strain in the soil communities.

### The Presence of Genetic Potential for Antibiotrophy in Soil Does Not Guarantee Its Expression

The relative abundance of the antibiotrophic gene *sadA* (Figure 1B) followed trends similar to those of *groL*. *sadA* was detected only when *Microbacterium* sp. C448 was present (i.e., when *groL* was detected), with relative abundances fluctuating between 0.3 ± 0.1 and 1.1 ± 0.4 *sadA* copies per 1,000 copies of *rrs* gene. More than just indicating that *Microbacterium* sp. C448 was initially the only host of *sadA* gene, it suggests that it kept its antibiotrophic genetic potential after its establishment in the SMZ-treated soils, whether or not manure was added. This agrees with the idea that the antibiotrophic function carried by *sadA* gene is probably useful in SMZ-polluted conditions.

Although previous studies have indicated that *sadA* is potentially mobile in other antibiotroph strains (Ricken et al., 2017; Reis et al., 2019), our results did not show any increase in the *sadA*/*groL* ratio (data not shown), suggesting an absence of horizontal gene transfer. This is consistent with the chromosomic localization of *sadA*, which makes its mobility *via* a horizontal gene transfer event unlikely (whole genome sequencing of *Microbacterium* sp. C448, Martin-Laurent et al., 2014).

The main outcome of *groL* and *sadA* gene sequence quantification is that installation of *Microbacterium* sp. C448 and its antibiotrophic gene *sadA* both succeed as long as they are under SMZ selection pressure. However, and since the gene’s presence does not guarantee its functional expression (Browning and Busby, 2004; Pietramellara et al., 2008), qPCR assessments were complemented with functional analyses to assess potential ^14^C-SMZ mineralization. As stated above, mineralization rates obtained for conditions without *Microbacterium* sp. C448 were low and did not exceed 3% in any of the soils (Figure 2). When *Microbacterium* sp. C448 was inoculated, a mineralization activity was observed for each soil, but it occurred only (in soils A, B, and D) or preferentially (in soil C) under manure spreading conditions (Figure 2). When the strain was applied simultaneously with manure, soils A, B, and D reached, respectively, 19.5 ± 1.2% (Figure 2), 15.3 ± 1.4% (Figure 2), and 5.6 ± 0.7% (Figure 2) of SMZ mineralization. Soil C was the only one where *Microbacterium* sp. C448 was able to express its mineralization potential without manure spreading (Figure 2). However, the mineralization rate was about half that with manure (5.5 ± 0.3% versus 12.6 ± 0.4%). Altogether, these results reveal that the presence of *Microbacterium* sp. C448 and its antibiotrophic gene *sadA* does not always result in effective antibiotrophy in SMZ-contaminated soils. In our experimental conditions, they reflect a modularity of the functional expression depending on the kind of soil and on manure addition what is commonly observed in particular for antibiotic degradation (Cycoń et al., 2019). This lack of mineralization did not seem to result from the absence of SMZ bioavailability. The quantification of the SMZ extractable fraction performed at the end of the incubation period indicates that the amount of bioavailable SMZ was not or only slightly affected by manure amendment (Table 1). *Microbacterium* sp. C448 thus potentially had access to as much SMZ with manure amendment as without. Moreover, the variation of free SMZ amount between soils does not seem to be correlated with the variation of mineralization between soils, since the soil C with the lowest SMZ concentration was not the one with the lowest degradation rate. Altogether, these observations provide a nuanced response to the hypothesis for how SMZ can give *Microbacterium* sp. C448 a selective advantage. Without manure, the absence of mineralization (or the drastically diminished mineralization compared to manure treatment for soil C) indicates that SMZ is not always essential as an exclusive dedicated nutrient source. We therefore investigated whether SMZ could also give this strain a selective advantage by its biocidal action to release ecological niches.

**TABLE 1:** Concentrations of extractable SMZ measured after 1 month of incubation in the four different types of soils treated with SMZ, amended or not with manure, and inoculated or not with *Microbacterium* sp. C448 (C448).

		Concentration of extractable SMZ (mg kg^–1^ dry soil)
Soil A	SMZ	54.1 ± 0.4
	SMZ × C448	54.4 ± 1.7
	SMZ × MANURE	45.4 ± 1.7
	SMZ × C448 × MANURE	34.6 ± 1.8
Soil B	SMZ	21.8 ± 2.7
	SMZ × C448	21.4 ± 0.8
	SMZ × MANURE	21.0 ± 2.6
	SMZ × C448 × MANURE	19.9 ± 1.5
Soil C	SMZ	0.9 ± 0.1
	SMZ × C448	0.6 ± 0.0
	SMZ × MANURE	1.1 ± 0.1
	SMZ × C448 × MANURE	0.5 ± 0.0
Soil D	SMZ	6.4 ± 0.2
	SMZ × C448	4.1 ± 0.1
	SMZ × MANURE	6.8 ± 0.1
	SMZ × C448 × MANURE	3.7 ± 0.1

### Antibiotrophy Efficiency Depends on SMZ Biocidal Impact

**FIGURE 3:**
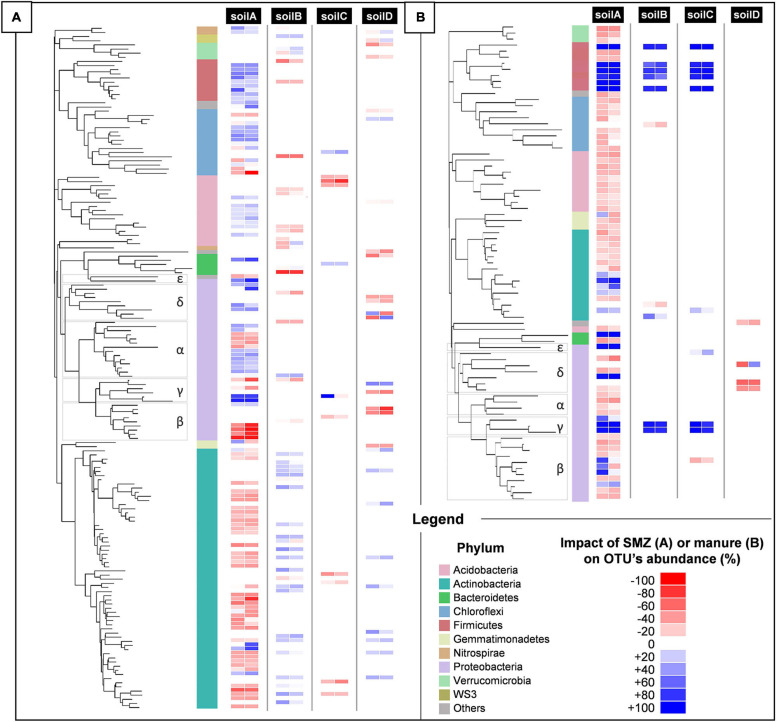
Phylogenetic placement of bacterial OTUs significantly impacted by SMZ **(A)** or manure spreading **(B)** in the four different types of soils, A, B, C, and D, after 1 month of incubation. Discriminant OTUs between treatments were assessed by ANOVA (*p* < 0.05). The phylum-level affiliation of each OTU is indicated by different colors in the left strip. For each OTU in each soil, the impact of SMZ **(A)** or manure spreading **(B)** is represented by a two-strip heatmap to distinguish conditions without (left strip) and with (right strip) manure spreading **(A)** or SMZ **(B)**. Red represents a negative impact of the treatment on the OTU abundance; blue, a positive impact. For each condition and OTU, the impact is reported as the difference in OTU abundance means between treated and non-treated samples divided by its maximum over the two conditions. Color intensities show the strength of the impact.

The biocidal impact of SMZ on soil communities was evaluated by 16S rDNA sequencing to assess the resulting changes in the bacterial and archeal community diversity and structure. From this sequencing, more than 23,100 partial 16S rDNA sequences were analyzed from each microcosm, accounting for 15,195 OTUs. The overall diversity indices (richness, equitability, and observed species) in the four tested soils were not significantly impacted by SMZ addition, whether or not manure and/or *Microbacterium* sp. C448 were added (Supplementary Table 4). This suggests no or only transient effects of SMZ on community diversity, but it does not exclude impacts on the members of the community. Further OTU-level ANOVAs revealed that about 165 of the studied OTUs were significantly affected by SMZ in at least one of the soils (Figure 3A). This limited impact is coherent with the previous study of Cleary et al. (2016) in which few OTUs were significantly affected by the input of SMZ into soils. The response of the bacterial community to SMZ varied between soils. The soil A community was clearly the most negatively impacted with 55 OTUs whose abundance was significantly reduced by the antibiotic, while the soil C community appeared to be the most robust one, with only eight OTUs negatively impacted (accounting for 14.1 and 2.3% of the bacterial community of untreated and non-amended soils, respectively). SMZ moderately affected soil B and D communities with, respectively, 15 and 20 OTUs with significantly reduced abundance (accounting for 5.3 and 2.7% of the bacterial community of untreated and non-amended soils, respectively). In parallel, the abundance of some OTUs was also increased, probably benefiting from the decrease in others. Pre-adaptation of bacterial communities to antibiotics could have been the main factor explaining the variations in the biocidal impact (Schmitt et al., 2004, 2005; Brandt et al., 2009). This was not the case here since the SMZ impact on the community of soil A was much stronger than on that of soil B, whereas they were exposed before the experiment to similar concentrations of SMZ (Supplementary Table 1). The second most probable factor explaining the variation of SMZ impact could be its bioavailability, since only the free fraction can be bioavailable for bacteria and exert its biocidal action. The SMZ quantification supports this hypothesis (Table 1). In the non-inoculated soils where no specific mineralization took place, the greatest SMZ available fraction was found in the most impacted soil (soil A, 54.1 ± 0.4% of SMZ available) and the lowest in the least impacted one (soil C, 0.9 ± 0.1%). The bioavailability of SMZ (and more generally of organic contaminants) relies on its intrinsic properties and on soil characteristics and especially clay content, organic carbon fraction, and pH (Supplementary Table 1). It is expected that their increases will reduce the bioavailability of SMZ in soil and therefore its biocidal activity (Gao and Pedersen, 2005; Lertpaitoonpan et al., 2009; Pinna et al., 2012). Our results are in line with this hypothesis since soil A, which hosted the most impacted community, and soil C, which hosted the least impacted one, exhibited, respectively, the lowest and highest values for clay content, organic carbon fraction, and pH (84, 5.2, and 5.6‰, and 360, 13, and 6.9‰, respectively).

Besides confirming that the biocidal activity of SMZ depends on the soil’s physical and chemical properties, microbial diversity and structure analyses suggest that ecological niches may have been freed after the reduction in abundance of some OTUs following the SMZ exposure. *Microbacterium* sp. C448 could have potentially colonized and used these unoccupied niches, thus facilitating its establishment in the soil community. This outcome supports the hypothesis of released niches, which could explain the crucial positive role of SMZ for *Microbacterium* sp. C448 establishment even when it does not use this antibiotic as a nutrient source. The lack (or limited level) of SMZ mineralization observed in the absence of manure spreading, although the antibiotic was available, may indicate a niche preference of the strain for readily available organic nutrients as already shown in other studies dealing with inoculation of xenobiotics degraders in soils (Benimeli et al., 2008). The degradation of antibiotics like many other synthetic organic contaminants can be arduous as the successive steps leading to their complete degradation can release intermediate transformation products that can be toxic for the degrading bacteria involved. For instance, sulfur dioxide, one dead-end product of SMZ mineralization (Ricken et al., 2017), is known for its antibacterial properties (Malwal et al., 2012). However, SMZ can constitute a nutritive source of prime choice for *Microbacterium* sp. C448, especially if the strain lacks niches to support its growth. The measurements of SMZ mineralization potential for the soil C fits this hypothesis. The slight mineralization of SMZ (5.5 ± 0.3%) and its reduced biocidal action on the bacterial community suggest that the release of trophic niches was insufficient to entirely support *Microbacterium* sp. C448 growth, which therefore used its antibiotic degradation in compensation.

### Antibiotrophy Efficiency Depends on Soil Permissiveness to Invasion of Manure-Originating OTUs and Resulting Trophic Competition

In soils spread with manure, antibiotic mineralization was enhanced although biocidal action of SMZ and therefore niche releases occurred in a similar way (Figure 2). This suggests that *Microbacterium* sp. C448 encountered difficulties in accessing trophic niches released by SMZ and had to rely on antibiotic degradation to become established.

We observed that adding manure significantly increased the alpha diversity of bacteria and archaea in each SMZ-treated soil, except for soil D (Supplementary Table 4). Phylogenetic diversity values increased by about 5.7, 5.2, and 7.2% in soils A, B, and C, respectively, with a respective gain of about 75, 68, and 116 observed OTUs. ANOVAs conducted on each OTU individually indicated that 79 of the 15,195 OTUs were significantly increased or decreased in response to manure application in at least one of these four soils (Figure 3B). In detail, soil A was the most affected, with a strong increase in abundance of 21 OTU (+80% of their abundance at least) and a slight decrease in abundance of 51 OTU (−60% at most). In the presence of manure, soils B and C presented increasing abundances only for eight and nine OTUs and decreasing abundances for one and two OTUs, respectively. Interestingly, a cluster of seven OTUs presented the same pattern in soils A, B, and C. They were below or near the detection limit without manure. Their abundance significantly increased in manure-spread soils. Some of these OTUs (two γ-Proteobacteria and five Firmicutes including four Clostridiales) are known as fecal markers (Hagey et al., 2019). Moreover, they were abundant in manure (Supplementary Table 5). According to Gravuer and Scow (2021), those observations are signs that these OTUs originate manure and are soil invaders. This supports the hypothesis that manure brought exogenous OTUs that compete with *Microbacterium* sp. C448 for establishment in soil. As stated above, *Microbacterium* sp. C448 seems to be a poor competitor in the studied soils. Simultaneous inputs of manure and the antibiotrophic strain could thus induce detrimental competition for *Microbacterium* sp. C448, in favor of using SMZ as a nutritional support of choice. This could thus explain why manure spreading led to a strong stimulation of SMZ mineralization, which reached 19.5, 15.3, and 12.6% in soils A, B, and C, respectively (Figure 2). On the contrary, the soil D community structure was only slightly affected by manure. Its diversity indices were not changed (Supplementary Figure 4) and the OTU-level analyses indicated that only four OTUs were significantly decreased, with no detected increase in individual OTU abundances (Figure 3B). Whether or not manure was added, soil D exhibited most of the seven OTUs belonging to the above cluster that was abundant in soils A, B, and C spread with manure (Supplementary Table 5). This initial presence in soil D might be due to its agronomical history, which involved regular application of manure as organic fertilizer (the latest occasion being 6 months before the experiment). Based on these results, we can hypothesize that the previous in-field manure exposure led to a pre-colonization of soil communities by manure OTUs (Gravuer and Scow, 2021), thus limiting further microbial disturbance in response to the experimental amendment of manure. It therefore suggests that most of the ecological niches made available by the biocidal effect of SMZ in this soil remained free for *Microbacterium* sp. C448, thus explaining, at least partially, the low SMZ mineralization rate (5.6 ± 0.7%) measured in soil D spread with manure (Figure 2).

## Conclusion

The sulfonamide-resistant function confers a selective advantage in competitive environments impacted by the biocidal action of SMZ. Our study strongly suggests that sulfonamide-degrading capacities could provide an additional selective advantage to sulfonamide-resistant bacteria by enhancing their dispersion potential. Besides eliminating sensitive trophic competitors, and so releasing niches, SMZ can constitute an exclusive trophic niche for sulfonamide-degraders. Our study shows that antibiotrophy is mainly beneficial when the SMZ biocidal activity on competitors is insufficient. Indeed, antibiotrophy promoted *Microbacterium* sp. C448 establishment when the impact of SMZ on soil microbial diversity and structure was low and when outsider competitors were added by manure amendment. The emergence of antibiotrophy among antibiotic-resistant bacteria might promote their dispersion and survival in the environment and thus might constitute a health concern. Antibiotrophy would be expected to provide an environmental benefit by contributing to the self-purifying of antibiotic-contaminated ecosystems. Yet, in our experiment, SMZ degradation was neither high nor automatic in soils with antibiotrophs. Further research is needed to better assess the role of the antibiotroph function in the dispersion of antibiotic-resistant bacteria in the environment. One of the possible ways to achieve this goal will be to compare the dispersion potential of the wild-type strain to that of a mutant unable to degrade and grow on SMZ.

## Data Availability Statement

The datasets presented in this study can be found in online repositories. The names of the repository/repositories and accession number(s) can be found below: https://www.ncbi.nlm.nih.gov/, SUB8712901. The latest accession in the SRA at NCBI number is: PRJNA684125.

## Author Contributions

LB, SP, MD-L, and FM-L contributed to the conception and design of the study. LB, NR, and MD-L performed the laboratory experiments. LB, MD-L, AS, and AM analyzed the results. LB, SP, and MD-L wrote the first draft of the manuscript. LP, ML, and PB-H developed the analytical methods for the SMZ quantification in soil and manure and analyzed the results. LP and PB-H prepared the soil and manure samples, and ML carried out the LC-MS analyses. All authors contributed to manuscript revision and read and approved the submitted version.

## Conflict of Interest

The authors declare that the research was conducted in the absence of any commercial or financial relationships that could be construed as a potential conflict of interest.

## Supplementary Material

Supplementary Figure 1 Experimental design of soil microcosm experiment with ^12^C-SMZ or ^14^C-SMZ, consisting of amendment or not with manure, treatment.

Supplementary Table 1 GPS coordinates and physical-chemical properties of the four different soils used in the study.

Supplementary Table 2 Primers and probe used in this study.

Supplementary Table 3 Mass balances of ^14^C-radioactivity in the four different soils treated with SMZ, amended or not with manure, and inoculated or not with *Microbacterium* sp. C448 (C448) obtained at the end of the month of incubation. Between 75% and 100% of the initially added radioactivity was retrieved. Results are expressed in percent of ^14^C-SMZ initially retrieved.

Supplementary Table 4 Alpha diversity indices of the four different soils amended or not with manure, treated or not with SMZ and inoculated or not with *Microbacterium* sp. C448 (C448).

Supplementary Table 5 Initial abundance and phylogeny (phylum, class, order) of the seven OTUs highly likely to come from manure in the four soils and in the manure.
