# Supplementary figures and images for: Antibiotrophy: Key Function for Antibiotic-Resistant Bacteria to Colonize Soils—Case of Sulfamethazine-Degrading Microbacterium sp. C448
Source: Front Microbiol. 2021 Mar 26;12:643087. doi: 10.3389/fmicb.2021.643087 (PMC8032547; doi:10.3389/fmicb.2021.643087)

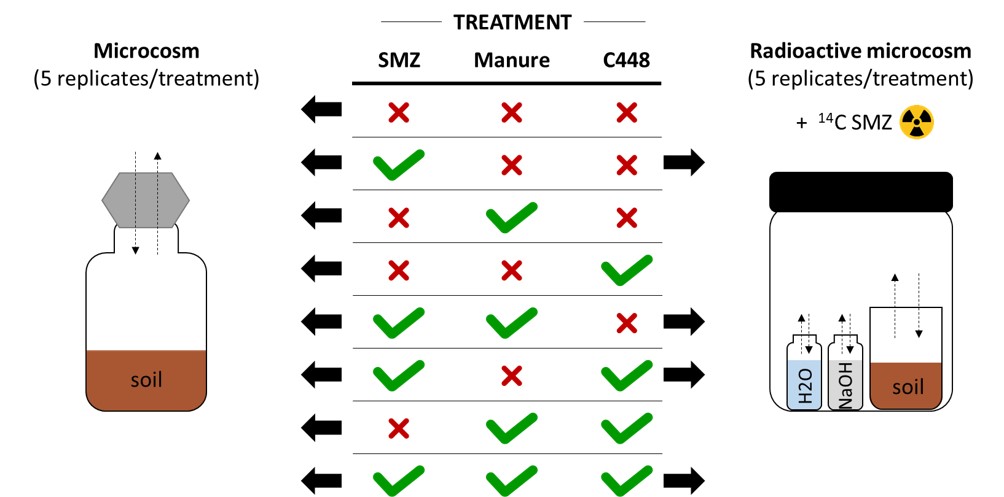

Supplement: Supplementary Figure 1 — Experimental design of soil microcosm experiment with 12C-SMZ or 14C-SMZ, consisting of amendment or not with manure, treatment. [file Image_1.jpeg]
