# Supplementary material for: Antibiotrophy: Key Function for Antibiotic-Resistant Bacteria to Colonize Soils—Case of Sulfamethazine-Degrading Microbacterium sp. C448
Source: Front Microbiol. 2021 Mar 26;12:643087. doi: 10.3389/fmicb.2021.643087 (PMC8032547; doi:10.3389/fmicb.2021.643087)
Supplement: Supplementary Table 1 — GPS coordinates and physical-chemical properties of the four different soils used in the study. [file Table_1.docx]

|  | |  |  |  | **Soil A** |  | **Soil B** |  | **Soil C** |  | **Soil D** |
| --- | --- | --- | --- | --- | --- | --- | --- | --- | --- | --- | --- |
| **GPS COORDINATES** | | | | | | | | | | | |
|  |  |  |  |  |  |  |  |  |  |  |  |
|  | Latitude |  | - |  | 47.239562 |  | 47.239506 |  | 47.231351 |  | 46.960258 |
|  | Longitude |  | - |  | 5.415079 |  | 5.416419 |  | 5.104477 |  | 5.483326 |
|  |  |  |  |  |  |  |  |  |  |  |  |
| **PHYSICAL-CHEMICAL PROPERTIES** | | | | | | | | | | | |
|  |  |  |  |  |  |  |  |  |  |  |  |
|  | Clay |  | ‰ |  | 84 |  | 215 |  | 360 |  | 146 |
|  | Fine silt |  | ‰ |  | 37 |  | 111 |  | 338 |  | 332 |
|  | Coarse silt |  | ‰ |  | 32 |  | 52 |  | 238 |  | 166 |
|  | Fine sand |  | ‰ |  | 117 |  | 147 |  | 37 |  | 64 |
|  | Coarse sand |  | ‰ |  | 731 |  | 474 |  | 28 |  | 292 |
|  |  |  |  |  |  |  |  |  |  |  |  |
|  | pH |  | - |  | 5.6 |  | 6.1 |  | 6.9 |  | 6.1 |
|  | Total carbonates |  | ‰ |  | 0 |  | 0 |  | 0 |  | 0 |
|  |  |  |  |  |  |  |  |  |  |  |  |
|  | Total organic carbon |  | ‰ |  | 5.2 |  | 9.9 |  | 13 |  | 10.4 |
|  | Organic matter |  | ‰ |  | 9 |  | 17.1 |  | 22.4 |  | 18 |
|  | Total nitrogen |  | ‰ |  | 0.54 |  | 1.15 |  | 1.37 |  | 1.26 |
|  | C/N ratio |  | - |  | 9.6 |  | 8.6 |  | 9.5 |  | 8.2 |
|  |  |  |  |  |  |  |  |  |  |  |  |
|  | Metson CEC |  | Me/kg |  | 41 |  | 112 |  | 165 |  | 79 |
|  |  |  |  |  |  |  |  |  |  |  |  |
|  | Water content at pF 2.7 |  | % |  | 6.8 |  | 14 |  | 21.4 |  | 18.3 |
|  | Water content at saturation |  | % |  | 28.5 |  | 37.1 |  | 39.1 |  | 40.8 |
|  |  |  |  |  |  |  |  |  |  |  |  |
|  | SMZ concentration |  | µg/kg |  | 0.63 |  | 0.43 |  | < 0.08 |  | < 0.08 |
|  |  |  |  |  |  |  |  |  |  |  |  |
