# Supplementary material for: Antibiotrophy: Key Function for Antibiotic-Resistant Bacteria to Colonize Soils—Case of Sulfamethazine-Degrading Microbacterium sp. C448
Source: Front Microbiol. 2021 Mar 26;12:643087. doi: 10.3389/fmicb.2021.643087 (PMC8032547; doi:10.3389/fmicb.2021.643087)
Supplement: Supplementary Table 2 — Primers and probe used in this study. [file Table_2.docx]

|  |  |  |  |  |  |  |
| --- | --- | --- | --- | --- | --- | --- |
| Target |  | Primer name |  | Primer sequence |  | References |
|  |  |  |  |  |  |  |
| *sadA* |  | sadA_F |  | CCG-GTA-CGG-ATG-ATG-ACT-CT |  | This study |
|  |  | sadA_R |  | GGG-ACC-ATA-GGC-GTG-AGA-TA |  |  |
|  |  |  |  |  |  |  |
| *groL* |  | D0321 |  | ACC-ATT-GGT-GAC-CTG-ATC |  | Links et al., 2018 |
|  |  | D0322 |  | CCT-TGT-CGA-AAC-GCA-TAC |  |  |
|  |  | C448_Probe |  | AAC-ACG-TTC-GGC-ACC-GAG-CT |  |  |
|  |  |  |  |  |  |  |
| *rrs*  (qPCR) |  | 341_F |  | CCT-ACG-GGA-GGC-AGC-AG |  | Watanabe et al., 2001 |
|  |  | 534_R |  | ATT-ACC-GCG-GCT-GCT-GGC-A |  |  |
|  |  |  |  |  |  |  |
| *rrs*  (sequencing) |  | Pro341 |  | CCT-ACG-GGN-BGC-ASC-AG |  | Takahashi et al., 2014 |
|  |  | Pro805R |  | GAC-TAC-NVG-GGT-ATC-TAA-TCC |  |  |
|  |  | adapter |  | Read1:  TCG-TCG-GCA-GCG-TCA-GAT-GTG-TAT-AAG-AGA-CAG  Read2:  GTC-TCG-TGG-GCT-CGG-AGA-TGT-GTA-TAA-GAG-ACA-G |  |  |
|  |  |  |  |  |  |  |

Links, M. G., Dumonceaux, T. J., McCarthy, L., Hemmingsen, S. M., Topp, E., Comte, A., et al. (2018). CaptureSeq: Capture-based enrichment of cpn60 gene fragments empowers pan-Domain profiling of microbial communities without universal PCR. *bioRxiv*, 492116. doi:10.1101/492116.

Watanabe, K., Kodama, Y., and Harayama, S. (2001). Design and evaluation of PCR primers to amplify bacterial 16S ribosomal DNA fragments used for community fingerprinting. J Microbiol Methods 44, 253–262. doi:10.1016/s0167-7012(01)00220-2.

Takahashi, S., Tomita, J., Nishioka, K., Hisada, T., and Nishijima, M. (2014). Development of a prokaryotic universal primer for simultaneous analysis of Bacteria and Archaea using next-generation sequencing. PLoS One 9, e105592. doi:10.1371/journal.pone.0105592.
