# Supplementary material for: Antibiotrophy: Key Function for Antibiotic-Resistant Bacteria to Colonize Soils—Case of Sulfamethazine-Degrading Microbacterium sp. C448
Source: Front Microbiol. 2021 Mar 26;12:643087. doi: 10.3389/fmicb.2021.643087 (PMC8032547; doi:10.3389/fmicb.2021.643087)
Supplement: Supplementary Table 3 — Mass balances of 14C-radioactivity in the four different soils treated with SMZ, amended or not with manure, and inoculated or not with Microbacterium sp. C448 (C448) obtained at the end of the month of incubation. Between 75% and 100% of the initially added radioactivity was retrieved. Results are expressed in percent of 14C-SMZ initially retrieved. [file Table_3.docx]

|  |  |  |  |  |  |
| --- | --- | --- | --- | --- | --- |
|  |  |  | **Non-extractable SMZ (%)** | **Extractable SMZ (%)** | **Mineralized SMZ (%)** |
|  |  |  |  |  |  |
| **Soil A** | SMZ |  | 59.4 ± 1.2 | 40.4 ± 1.2 | 0.2 ± 0.01 |
|  | SMZ x C448 |  | 56.8 ± 1.4 | 43.0 ± 1.4 | 0.2 ± 0.03 |
|  | SMZ x MANURE |  | 56.7 ± 1.5 | 43.1 ± 1.5 | 0.2 ± 0.02 |
|  | SMZ x C448 x MANURE |  | 56.8 ± 1.5 | 22.7 ± 0.9 | 20.5 ± 1.4 |
|  |  |  |  |  |  |
| **Soil B** | SMZ |  | 81.9 ± 3.2 | 15.9 ± 3.7 | 2.2 ± 0.5 |
|  | SMZ x C448 |  | 81.0 ± 0.5 | 16.2 ± 0.5 | 2.8 ± 0.1 |
|  | SMZ x MANURE |  | 83.0 ± 0.4 | 14.9 ± 0.4 | 2.1 ± 0.2 |
|  | SMZ x C448 x MANURE |  | 73.7 ± 1.8 | 9.,6 ± 1.2 | 16.7 ± 0.7 |
|  |  |  |  |  |  |
| **Soil C** | SMZ |  | 92.8 ± 0.4 | 3.7 ± 0.2 | 3.4 ± 0.3 |
|  | SMZ x C448 |  | 89.2 ± 0.3 | 3.4 ± 0.3 | 7.3 ± 0.4 |
|  | SMZ x MANURE |  | 92.7 ± 0.4 | 4.4 ± 0.3 | 2.9 ± 0.2 |
|  | SMZ x C448 x MANURE |  | 81.4 ± 0.8 | 3.4 ± 0.4 | 15.2 ± 0.,4 |
|  |  |  |  |  |  |
| **Soil D** | SMZ |  | 87.9 ± 0.4 | 9.6 ± 0.,4 | 2.5 ± 0.1 |
|  | SMZ x C448 |  | 87.5 ± 0.3 | 9.6 ± 0.3 | 2.9 ± 0.1 |
|  | SMZ x MANURE |  | 88.0 ± 0.3 | 10.1 ± 0.3 | 1.8 ± 0.1 |
|  | SMZ x C448 x MANURE |  | 86.6 ± 0.4 | 9.0 ± 0.4 | 4.5 ± 0.5 |
|  |  |  |  |  |  |
