# Supplementary material for: Antibiotrophy: Key Function for Antibiotic-Resistant Bacteria to Colonize Soils—Case of Sulfamethazine-Degrading Microbacterium sp. C448
Source: Front Microbiol. 2021 Mar 26;12:643087. doi: 10.3389/fmicb.2021.643087 (PMC8032547; doi:10.3389/fmicb.2021.643087)
Supplement: Supplementary Table 4 — Alpha diversity indices of the four different soils amended or not with manure, treated or not with SMZ and inoculated or not with Microbacterium sp. C448 (C448). [file Table_4.docx]

|  |  | **Treatment** |  |  |  | **PD whole tree** |  | **Equitability** |  | **Observed species** |  | **Seqs/Sample** |
| --- | --- | --- | --- | --- | --- | --- | --- | --- | --- | --- | --- | --- |
|  |  |  |  |  |  |  |  |  |  |  |  |  |
| **Soil A** | **Manure** | C448 |  | **SMZ** |  | 211 ± 4 |  | 0.824 ± 0.002 |  | 3338 ± 42 |  | 25700 |
|  |  |  |  | **Ø SMZ** |  | 192 ± 6 |  | 0.812 ± 0.01 |  | 3100 ± 115 |  |  |
|  |  |  |  |  |  |  |  |  |  |  |  |  |
|  |  | **Ø** C448 |  | **SMZ** |  | 205 ± 6 |  | 0.806 ± 0.015 |  | 3224 ± 59 |  |  |
|  |  |  |  | **Ø SMZ** |  | 198 ± 2 |  | 0.819 ± 0.002 |  | 3156 ± 32 |  |  |
|  |  |  |  |  |  |  |  |  |  |  |  |  |
|  | **Ø Manure** | C448 |  | **SMZ** |  | 195 ± 5 |  | 0.83 ± 0.002 |  | 3224 ± 56 |  |  |
|  |  |  |  | **Ø SMZ** |  | 189 ± 4 |  | 0.822 ± 0.001 |  | 3042 ± 21 |  |  |
|  |  |  |  |  |  |  |  |  |  |  |  |  |
|  |  | **Ø** C448 |  | **SMZ** |  | 192 ± 3 |  | 0.826 ± 0.001 |  | 3158 ± 31 |  |  |
|  |  |  |  | **Ø SMZ** |  | 189 ± 3 |  | 0.824 ± 0.003 |  | 3093 ± 36 |  |  |
|  |  |  |  |  |  |  |  |  |  |  |  |  |
|  |  |  |  |  |  |  |  |  |  |  |  |  |
| **Soil B** | **Manure** | C448 |  | **SMZ** |  | 197 ± 4 |  | 0.788 ± 0.005 |  | 3117 ± 39 |  | 24500 |
|  |  |  |  | **Ø SMZ** |  | 197 ± 8 |  | 0.799 ± 0.01 |  | 3145 ± 92 |  |  |
|  |  |  |  |  |  |  |  |  |  |  |  |  |
|  |  | **Ø** C448 |  | **SMZ** |  | 198 ± 5 |  | 0.798 ± 0.006 |  | 3162 ± 54 |  |  |
|  |  |  |  | **Ø SMZ** |  | 200 ± 5 |  | 0.798 ± 0.008 |  | 3209 ± 74 |  |  |
|  |  |  |  |  |  |  |  |  |  |  |  |  |
|  | **Ø Manure** | C448 |  | **SMZ** |  | 184 ± 3 |  | 0.792 ± 0.004 |  | 3033 ± 39 |  |  |
|  |  |  |  | **Ø SMZ** |  | 189 ± 6 |  | 0.798 ± 0.01 |  | 3095 ± 85 |  |  |
|  |  |  |  |  |  |  |  |  |  |  |  |  |
|  |  | **Ø** C448 |  | **SMZ** |  | 189 ± 6 |  | 0.8 ± 0.007 |  | 3090 ± 62 |  |  |
|  |  |  |  | **Ø SMZ** |  | 191 ± 5 |  | 0.789 ± 0.01 |  | 3144 ± 86 |  |  |
|  |  |  |  |  |  |  |  |  |  |  |  |  |
|  |  |  |  |  |  |  |  |  |  |  |  |  |
| **Soil C** | **Manure** | C448 |  | **SMZ** |  | 196 ± 1 |  | 0.819 ± 0.003 |  | 3132 ± 19 |  | 28300 |
|  |  |  |  | **Ø SMZ** |  | 190 ± 3 |  | 0.82 ± 0.004 |  | 3056 ± 48 |  |  |
|  |  |  |  |  |  |  |  |  |  |  |  |  |
|  |  | **Ø** C448 |  | **SMZ** |  | 191 ± 3 |  | 0.812 ± 0.012 |  | 2992 ± 81 |  |  |
|  |  |  |  | **Ø SMZ** |  | 193 ± 5 |  | 0.814 ± 0.006 |  | 3083 ± 66 |  |  |
|  |  |  |  |  |  |  |  |  |  |  |  |  |
|  | **Ø Manure** | C448 |  | **SMZ** |  | 182 ± 5 |  | 0.809 ± 0.007 |  | 2938 ± 46 |  |  |
|  |  |  |  | **Ø SMZ** |  | 177 ± 4 |  | 0.814 ± 0.006 |  | 2933 ± 75 |  |  |
|  |  |  |  |  |  |  |  |  |  |  |  |  |
|  |  | **Ø** C448 |  | **SMZ** |  | 179 ± 4 |  | 0.815 ± 0.004 |  | 2960 ± 55 |  |  |
|  |  |  |  | **Ø SMZ** |  | 178 ± 3 |  | 0.815 ± 0.003 |  | 2967 ± 24 |  |  |
|  |  |  |  |  |  |  |  |  |  |  |  |  |
|  |  |  |  |  |  |  |  |  |  |  |  |  |
| **Soil D** | **Manure** | C448 |  | **SMZ** |  | 241 ± 3 |  | 0.858 ± 0.003 |  | 3933 ± 30 |  | 23100 |
|  |  |  |  | **Ø SMZ** |  | 237 ± 5 |  | 0.852 ± 0.003 |  | 3830 ± 62 |  |  |
|  |  |  |  |  |  |  |  |  |  |  |  |  |
|  |  | **Ø** C448 |  | **SMZ** |  | 238 ± 2 |  | 0.853 ± 0.005 |  | 3857 ± 35 |  |  |
|  |  |  |  | **Ø SMZ** |  | 242 ± 3 |  | 0.854 ± 0.003 |  | 3887 ± 35 |  |  |
|  |  |  |  |  |  |  |  |  |  |  |  |  |
|  | **Ø Manure** | C448 |  | **SMZ** |  | 235 ± 3 |  | 0.858 ± 0.003 |  | 3866 ± 39 |  |  |
|  |  |  |  | **Ø SMZ** |  | 238 ± 4 |  | 0.856 ± 0.003 |  | 3885 ± 81 |  |  |
|  |  |  |  |  |  |  |  |  |  |  |  |  |
|  |  | **Ø** C448 |  | **SMZ** |  | 233 ± 2 |  | 0.852 ± 0.006 |  | 3812 ± 32 |  |  |
|  |  |  |  | **Ø SMZ** |  | 239 ± 3 |  | 0.858 ± 0.003 |  | 3870 ± 33 |  |  |
|  |  |  |  |  |  |  |  |  |  |  |  |  |
