# Supplementary material for: Antibiotrophy: Key Function for Antibiotic-Resistant Bacteria to Colonize Soils—Case of Sulfamethazine-Degrading Microbacterium sp. C448
Source: Front Microbiol. 2021 Mar 26;12:643087. doi: 10.3389/fmicb.2021.643087 (PMC8032547; doi:10.3389/fmicb.2021.643087)
Supplement: Supplementary Table 5 — Initial abundance and phylogeny (phylum, class, order) of the seven OTUs highly likely to come from manure in the four soils and in the manure. [file Table_5.docx]

|  |  | | | | |  |  | | |
| --- | --- | --- | --- | --- | --- | --- | --- | --- | --- |
|  | **Abundance** | | | | |  | **Phylogeny** | | |
|  |  |  |  |  |  |  |  |  |  |
|  | **Soil A** | **Soil B** | **Soil C** | **Soil D** | **Manure** |  | **Phylum** | **Class** | **Order** |
|  |  |  |  |  |  |  |  |  |  |
|  |  |  |  |  |  |  |  |  |  |
| OTU1 | 0.4  ± 0.5 | 1.4  ± 1.7 | 14 ± 23 | 120 ± 75 | 299  ± 55 |  | Firmicutes | Bacilli | Turicibacterales |
|  |  |  |  |  |  |  |  |  |  |
| OTU2 | 0.2  ± 0.3 | 5.2  ± 4.3 | 102  ± 163 | 514 ± 196 | 702 ± 146 |  | Firmicutes | Clostridia | Clostridiales |
|  |  |  |  |  |  |  |  |  |  |
| OTU3 | 4  ± 1.2 | 7.6  ± 4.7 | 38 ± 58 | 218 ± 78 | 328  ± 62 |  | Firmicutes | Clostridia | Clostridiales |
|  |  |  |  |  |  |  |  |  |  |
| OTU4 | 2.2  ± 0.6 | 1.8  ± 1 | 20  ± 31 | 90  ± 39 | 165  ± 36 |  | Firmicutes | Clostridia | Clostridiales |
|  |  |  |  |  |  |  |  |  |  |
| OTU5 | 6  ± 2.8 | 7.2  ± 1.8 | 29 ± 47 | 122  ± 41 | 128 ± 25 |  | Firmicutes | Clostridia | Clostridiales |
|  |  |  |  |  |  |  |  |  |  |
| OTU6 | 0.2  ± 0.3 | 6.4  ± 9.4 | 0.2  ± 0.3 | 670  ± 1071 | 2531  ± 253 |  | Proteobacteria | γ-Proteobacteria | Pseudomonadales |
|  |  |  |  |  |  |  |  |  |  |
| OTU7 | 0.4  ± 0.5 | 3  ± 4.4 | 0.2  ± 0.3 | 440 ± 704 | 1577  ± 120 |  | Proteobacteria | γ-Proteobacteria | Pseudomonadales |
|  |  |  |  |  |  |  |  |  |  |
